# Feeling safe, feeling connected: aesthetic and polyvagal approaches to dementia care

**DOI:** 10.3389/frdem.2025.1735205

**Published:** 2026-01-12

**Authors:** Sarah Fox, Jennie Davies, Robyn Dowlen, John Keady, James Thompson, Ruth Watson

**Affiliations:** 1 NIHR Applied Research Collaboration, Manchester, United Kingdom; 2 Global Brain Health Institute, Trinity College Dublin, Dublin, Ireland; 3 Greater Manchester Mental Health NHS Foundation Trust, Manchester, United Kingdom; 4 Research Centre for Arts and Wellbeing, Health Research Institute, Faculty of Health, Social Care and Medicine, Edge Hill University, Ormskirk, United Kingdom; 5 Division of Nursing, Midwifery and Social Work, The University of Manchester, Manchester, United Kingdom; 6 School of Arts Languages and Cultures, The University of Manchester, Manchester, United Kingdom

**Keywords:** dementia, aesthetics, Polyvagal Theory, neuroaesthetics, care

## Abstract

This perspective brings together authors from care aesthetics, dementia studies, mental health nursing, and clinical psychology to explore how aesthetics and Polyvagal Theory intersect in dementia care. Across these fields, there is growing recognition that wellbeing is shaped not only by clinical interventions but also by the subtle, embodied cues that create a sense of safety, connection, and belonging for patients. Concepts such as aesthetic care, in-the-moment practices, and everyday aesthetics emphasize how lived experience and wellbeing is grounded in the sensory and relational details of everyday life. In parallel, Polyvagal Theory provides a psychophysiological framework for understanding how people respond to such cues through the process of threat detection, co-regulation, and social engagement. By placing these perspectives side-by-side, we explore the currently untapped benefits of developing a cross disciplinary therapeutic toolkit for clinicians working with people living with dementia. Looking ahead, integrating aesthetics and Polyvagal-informed approaches could reshape dementia care into a practice that values safety, connection, and meaning as core clinical outcomes. Although further research is needed to translate this integrated model into practice, the work of the authorship in both research and clinical practice with people with dementia illustrate that such approaches are already ongoing and can bring tangible benefits for several stakeholders, including people living with dementia.

## 1 Introduction

The concepts of aesthetics, Polyvagal Theory, moments, and wellbeing have each attracted growing interest across diverse fields, yet they are rarely considered together. In this perspective paper, we bring researchers and practitioners working in care aesthetics, dementia studies, mental health nursing, and clinical psychology into a dialogue to offer a fresh perspective on how fleeting yet powerful moments of connection and meaning shape, psychological and physiological wellbeing for people living with dementia. Each of these disciplines brings a distinctive perspective, whether this is on the sensory and relational qualities of care, the lived experience of aging, cognitive change and wellbeing, the embodied process of affect regulation or the neurophysiological correlates of connection and safety. By bringing these topics into dialogue, we seek to extend current understandings and open new conceptual ground for thinking about how everyday encounters can be harnessed to foster safety, connection, and wellbeing for people with dementia.

We begin by illustrating this point with a story collected as part of Fox et al. (2025)'s study which explored how people living with dementia and their carers created, experienced and interpreted aesthetic moments in their day-to-day lives:

During an interview with a gentleman living with dementia and his wife discussing his recent hospital stay, he described the experience in an unexpected way, he called it “homely.” When asked to explain, his wife stepped in: “*The nurses were all dead friendly and chatty, and gave you a bit of stick, didn't they?”*, at this he smiled and laughed.

A friendly face and gentle teasing may never appear on a care plan, yet in that moment, it was exactly what he needed. His wife had already expressed that he was historically wary of hospitals after a bad experience; this time, warmth and humor, tailored to his needs, made him feel safe in an otherwise anxiety-provoking environment.

This example highlights an aspect of dementia care which is often overlooked: the safety and connection provided by an attuned, embodied relationship between care provider, care setting and patient. A recent letter to the editor in the International Journal of Geriatric Psychiatry (Corpuz, 2025), eloquently argues the need for an approach to care that recognizes how aesthetic experiences shape wellbeing and reminds us that “*dignity is preserved not only through medical treatment but also through everyday beauty and connection”*.

We suggest that a framework which combines awareness of the unique experiences and environments which bring aesthetic comfort with existing psychotherapeutic approaches to safety and wellbeing, such as Polyvagal therapy (Porges, 2022), offers a novel approach through which aesthetic awareness may be integrated into everyday life and clinical practice. Indeed, we suggest that combining these fields may provide a starting point in the development of a novel systematic Everyday Aesthetic Care Model (EACM) for people living with dementia as proposed by Corpuz (2025).

To build our argument we will first discuss the fields of aesthetics and Polyvagal Theory separately, before moving to consider how aspects of both approaches are already being used, informally in clinical dementia settings, finally drawing conclusions and suggesting next steps.

Throughout this paper we will be using terminology specific to the fields of aesthetics, person-centered dementia care and Polyvagal Theory, please see “Box1” for a glossary of terms.

## 2 Aesthetics in dementia care

Aesthetics has long been a part of care practice, from the everyday practices of displaying personalized images and photos on residents' doors in care homes, to the aesthetic of care observable in the way support workers deliver respectful, entuned intimate care to an anxious patient. However, while this aspect of care practice is often present, it is rarely explicitly taught, recognized or formally evaluated.

Jones et al. (2025) highlights the importance of aesthetics in her observational study of residents on a dementia inpatient ward, vividly describing the sensory and embodied relational care practice she observes alongside the contribution of the atmosphere on the ward. The importance of the aesthetics in care is further discussed by Kindell et al. (2025) in her work developing a practice model for health care support workers. Here she observes how support workers draw on a spectrum of senses and embodied practices to become co-present with the people they care for. These practices include bodily forms of connection, for example kneeling, holding hands, linking arms; vocal forms, including, adjusting tone and volume of voice, humming, singing and whispering; and sensory awareness such as, listening, watching etc.

Fox et al. (2025)'s work on everyday aesthetics in dementia care resonates with both the aesthetic care literature and a wider body of “in the moment” research in dementia studies, which examines lived experiences at the micro-temporal scale, fleeting, embodied interactions that can nevertheless carry deep personal significance. Such studies show that moments of recognition, sensory enjoyment, or shared humor can act as anchor points for well-being, even in the face of cognitive decline (Fox et al., 2025; Fu et al., 2021; Lai Kwan et al., 2019; Mittner, 2021; Dowlen et al., 2024; Keady et al., 2022; Kindell et al., 2018). Crucially, the value of these moments is not solely in their emotional valence at the time, but in how they contribute to ongoing narratives of self and relationship, sustaining a sense of continuity and belonging. Such research may be thought of as drawing on the aesthetic qualities of moments and how this quality affects people living with dementia.

Taken together, we suggest that meaningful aesthetic moments are integral to living well with dementia. Moments which hold a positive aesthetic value for the patient, for example interaction with a treasured object or beautiful environment, will have an equally positive impact on their wellbeing, while those which are viewed negatively, for example a dismissive social interaction or unpleasant sound, will contribute to feelings of disconnect and discomfort. In clinical contexts, this has significant implications: it invites us to view “good care” as not only the timely administration of medication or adherence to procedural safety, but also the cultivation of moments borne from environments and interactions that cue safety, belonging, and connection based on the patient's unique aesthetic preferences. It requires an ongoing attunement to the present moment, how people with dementia's aesthetic preferences may have been shaped by past experiences but also their changing relationship with the sensory world.

Such aesthetic practices are often disregarded by care workers themselves and described as “*just the little things”*. However, experimental neuroscientific and psychotherapeutic research is starting to uncover just how important aesthetic aspects of care may be to the long-term wellbeing of people with dementia (Fox et al., 2025; Kindell et al., 2018; Jones et al., 2025; Fleetwood-Smith et al., 2022).

## 3 Polyvagal theory as an approach to aesthetic dementia care

Dementia is an umbrella term describing a set of conditions which impact an individual's cognitive abilities. Symptoms range from memory impairment to changes in linguistic, sensory, higher processing and problem-solving abilities. While these symptoms are typically underpinned by progressive build-up of neural pathology, the extent and distribution of this pathology is often not well correlated with the severity of clinical symptoms. Indeed, population and community-based studies have shown a disconnect between pathological burden and cognitive symptoms, suggesting a role for alternative “modulating factors” in determining when and to what extent an individual experiences cognitive decline (Wharton et al., 2023).

One potential modulating factor, which might impact clinical presentation, is the subjective emotional and mental experiences of the individual living with dementia. Indeed, the late professor Kitwood (1997), suggested that the clinical symptoms of dementia progress based on both changes in brain pathology and an individual's psychosocial environment. Kitwood argued that actions which undermine an individual's personhood and wellbeing, which he termed malignant social psychology, could lead to more rapid symptomatic decline. Alternatively, positive person work, intended to fulfill the psychological needs of a person living with dementia, enhancing their personhood and wellbeing, would support them to remain independent for longer.

While Kitwood did not suggest neurological mechanisms through which positive person work could enhance an individual's wellbeing, his framework does point toward experiences where psychology and neuroscience might interact. Specifically, positive person work highlights interactions which encourage feelings of safety and connection, while simultaneously reducing experiences which trigger social disconnect and threat perception. This idea shares conceptual crossover with an area of study known as Polyvagal Theory (Porges, 2022), also described as the “science of safety.”

Polyvagal Theory was developed by neuroscientist Stephen Porges and focuses on the body's responses to internal, social and environmental cues of safety and threat. Porges argues that detection of bodily, social and environmental cues of threat and safety, occurring below conscious awareness, lead to state changes in the autonomic nervous system. He states that the autonomic nervous system is divided into three branches which cycle in a predictable hierarchical order. Each branch has recognizable physiological correlates and functions in a unique manner, allowing us to carry out our everyday tasks. The ventral branch being the most evolutionarily recent is associated with homeostasis, social connection and feelings of safety. The sympathetic branch can either be associated with activation, allowing us to engage in energy-intensive daily tasks or, in times of threat, it is associated with the fight or flight response. Finally, the most primitive branch the dorsal vagus, is linked with periods of calm and rest or, in threatening situations, this may lead to a “shut down” or “freeze” response (Porges, 2022, 2025).

In collaboration with clinical social worker Dana (2023), Polyvagal Theory has been translated into practice. This practice focuses on supporting patients to recognize unconscious shifts in their autonomic state, to gain understanding of how their brains interpret these shifts and to recognize which experiences precipitate the shifts. Circling back to Kitwood's theories, the experiences which Dana and Porges suggest are important in signaling safety to the autonomic nervous system, resonate with Kitwood's examples of actions reflective of positive personhood, these also share conceptual crossover with the field of aesthetic and “in the moment” care, for example:

- Co-regulation: Polyvagal Theory explores how a sense of therapeutic presence can facilitate safety, this may be achieved through social safety cues, such as eye contact, facial expression, touch, gesture or prosodic (calming) vocalizations. In a similar vein, Kitwood argues the importance of eye contact to acknowledge a person's unique presence, tone of voice and emotional resonance to communicating respect, and physical presence, closeness and touch to provide reassurance. In the field of care aesthetics this is referred to as co-presence and is highlighted as an important aspect of “good” aesthetic care (Kindell et al., 2025; Jones et al., 2025).
- Glimmers: Dana (2024) suggests that it's important to find experiences which can bring a patient back to a state of autonomic safety, she provides examples including; noticing places which bring you a sense of regulation and connection, finding objects that are important to you, observing soundscapes and music that engenders a feeling of safety and “remembering safety” including mentally revisiting moments of previous safety. Dana names these moments glimmers, a term that captures their fleeting yet potent capacity to evoke connection and calm. Kitwood suggest that related experiences, such as music, familiar objects, soothing environments and access to nature also play an important role in positive person work. Glimmers, also share conceptual crossover with the concept of meaningful moments and everyday aesthetics (Fox et al., 2025; Fleetwood-Smith et al., 2022; Saito, 2010; Leddy, 2015).

Taken together, Kitwood's concept of positive person work, Polyvagal Theory's emphasis on co-regulation, and Dana's description of glimmers can be seen to share crossover with theories of aesthetic and “in the moment” care. Specifically, each highlights the importance of subtle, sensory, embodied, meaningful moments and actions, focusing on their capacity to affirm personhood, improve wellbeing and safety for people living with dementia.

Therefore, while research in aesthetics highlights the importance of everyday experiences and interactions for people living with dementia, Polyvagal Theory and therapy provides a tested therapeutic framework upon which to develop a novel clinical aesthetic care model. We believe that such an approach, combining knowledge from aesthetics', care aesthetics, in the moment practices, Polyvagal Theory and neuroaesthetics into a cross disciplinary model, would be greater than the sum of its parts (Fox et al., 2025; Dowlen et al., 2024; Jones et al., 2025; Keady et al., 2022; Kindell et al., 2025; Mittner, 2021; Richardson and Campbell, 2024; Fleetwood-Smith et al., 2022; Dana, 2023; Porges, 2022, 2025). Indeed, we will now explore how these fields are already being informally combined in current clinical practice.

## 4 Reflections from practice

Co-authors Ruth Watson and Jennie Davies, both work clinically with people living with dementia and draw on aspects of Polyvagal Theory, including triggers and glimmers, to support the people living with dementia in their care. Watson is a clinical psychologist working in an NHS inpatient ward in Northwest England. Her work focuses on supporting individuals presenting with the most complex behaviors that challenge, which cannot be managed safely in the community. The acute nature of this environment means that service users frequently display high levels of agitation and distress. The aim of an admission is to reduce this distress and support staff to manage any BtC in the least restrictive way. Her multi-disciplinary team works closely to observe and understand how service users present on the ward, and how they interact with the environment.

Polyvagal Theory offers Watson a useful framework to consider how the person with dementia may respond to the ward environment, specifically in identifying the ways a patient's autonomic nervous system reacts to triggering experiences and regulates their response, for example autonomic states of fight/flight or freeze/withdraw. Ruth's team have access to background information about the person living with dementia, which can help them to consider the patient's earlier life experiences and what may constitute a trigger. Where possible, the team works to predict and minimize the presence of triggers (e.g. approaching from behind), this reduces the likelihood of the person's threat system being activated. However, the requirement for staff to undertake personal care (e.g. washing, dressing) presents an experience for the patient which is highly likely to trigger distress and cannot be completely avoided. Therefore, in these situations the team will often use a patient's history and information from family carers to identify and use glimmers as a guide to help soothe distress. Team formulation is a key opportunity to collate what the team have observed to work well, and also a valuable space to offer ways to understand behaviors. When patients place themselves on the floor, this can often be labeled as “behavioral,” however this framework would help us to consider the freeze / withdraw response, which can therefore inform a more compassionate response.

Ruth further states that,

“*Developing a consistent team approach is a core aim, which includes the most effective methods of co-regulation that staff have observed—usually tone of voice, facial expression and gestures/touch. It is also important that staff are supported to regulate their own nervous system, which can be difficult when working in an acute environment. This forms a key message within staff training and team formulation sessions*.”

Davies, a clinical psychologist working in an NHS community mental health team (CMHT) in North-West England, emphasizes the role of connection in improving quality of life for people with dementia. Cognitive changes in dementia often lead to isolation, or a sense of disconnection (Kitwood, 1997), clinically this is seen to impact on the patient's sense of everyday safety. While Davies may not explicitly discuss Polyvagal Theory with her service users or their care team, she often finds it useful to hold in mind the theories' therapeutic ideas with the aim of supporting service users to enter a state of safety and connectedness. Her team often shares a simple message that resonates with both staff and service users: “Have things and people around you that make you feel safe.” She reflects:

“*In clinical practice, it can be easy to get drawn into the pressure to “fix,” but what does this mean in the context of dementia care? It is important that we do not forget the power of meaningful moments and the power of co-regulation. In a recent discussion with a colleague, I was struck by the power they felt through simply making meaningful eye contact with certain individuals. By pulling together a formulation that highlights key elements of an individuals' life, along with what they have been able to communicate to their care partners, we are able to hypothesize how they might be feeling currently. This can then lead to best guesses as to what they may need in their environment to support them to maintain a sense of safety and personhood. Pulling together this information and reflecting on how and why a seemingly small moment is so important to an individual reinforces the subtle actions the care team can engage in, not as a way to “fix” but rather as a way to be with, which is arguably more important in dementia care*.”

Davies is also looking to explore structured interventions with the capacity to concurrently scaffold aesthetic moments, decreasing unintentional malignant psychology and fostering chances of glimmers. She suggests that one such intervention may be

“*Co-development of a ‘life story book' alongside the person living with dementia. This activity provides an opportunity to feel heard as a person with a history and preferences, and to connect in the moment on a meaningful level. It fosters co-regulation and positive person work during the development of the book, but also provides a tool for the individual to take forward to scaffold more glimmers and positive experiences in the future alongside their care partners. A physical book that holds key information the individual chooses to share about themselves can itself provide connection and glimmers, e.g. engaging with photos in the book. This tool can also allow care partners who do not know the individual well (e.g. agency staff members, or during an acute hospital admission) to have easy access to highly personalized information*.”

These examples show that, although often used informally, clinical practitioners are already engaging with concepts behind Polyvagal Theory and aesthetics to inform the care they provide for people living with dementia. While Polyvagal Theory provides a therapeutic model, the inputs to this model (co-presence, triggers and glimmers) are aesthetic and are thus best understood through an aesthetic lens. This suggests that an a care model which combines Polyvagal Theory with aesthetics, will best support the development of an expanded therapeutic toolbox for dementia care which is more than the sum of its parts.

## 5 Conclusion, aesthetic futures

We believe that the fields of aesthetics, “in the moment” care practices and Polyvagal Theory are, in many ways, speaking the same language. When brought together, each contributes unique insights into how to create positive and meaningful environments that support people with dementia to retain a level of autonomy and personal independence. Based on the observation of conceptual overlap between these fields, the first step toward developing an Everyday Aesthetic Care Model (EACM) (Corpuz, 2025) will be to perform a concept synthesis which systematically combines knowledge across these fields (Jabareen, 2009). Such an analysis will identify related concepts across fields, while also showing where the fields diverge, highlighting where knowledge from one field may extend another.

Looking ahead, integrating aesthetics and Polyvagal-informed approaches could provide a framework to reshape dementia care into a practice that values safety, connection, and meaning as core clinical outcomes. Future care models should move beyond the binary of “managing symptoms” vs. “providing activities,” instead embedding aesthetic attunement into every layer of service delivery, from ward design, to staff training, to care planning. This would mean recognizing small moments and sensory cues not as “extras” but as essential aspects of a therapeutic approach to wellbeing: the rhythm of speech, the type of lighting, or the micro-interactions that signal belonging. Such a model may draw from the work of Kindell et al. (2025), highlighting care practices, environments and infrastructures which encourage entuned aesthetic approaches to care. In such a model, the environment and interpersonal interactions would be treated with the same clinical rigor as medication, each forming part of a therapeutic landscape of care.

At the same time, we will need to develop new methods to capture and evaluate these aesthetic and Polyvagal-informed dimensions of care. Traditional outcome measures focused on cognition or functional independence often miss the subtle but profound impact of micro-moments of safety and connection. Future studies could use participatory and embodied methodologies to better understand how people with dementia experience glimmers, triggers, and aesthetic attunement in everyday contexts. Alongside such studies, methods which monitor biological signals indicative of autonomic activation may also provide quantitative measures of everyday meaning and connection (Fu et al., 2021; Lai Kwan et al., 2019). By doing so, dementia care could evolve toward a practice that does not simply extend life, but enriches it, sustaining personhood, relationships, and the felt sense of safety that allows people to live well with dementia.

## Data Availability

The original contributions presented in the study are included in the article/supplementary material, further inquiries can be directed to the corresponding author.

## Box 1

BOX 1 Glossary

- Aesthetic care: care practices that attend to sensory and embodied experience, such as tone of voice, gentle touch, or respectful gestures. Rather than being incidental, these practices are central to how safety and dignity are experienced (Thompson, 2022; Jones et al., 2025; Kindell et al., 2025).
- Co-regulation (polyvagal theory): the process through which one person's presence, gestures, or voice helps another's nervous system return to a state of safety. Co-regulation underpins relational care and is a cornerstone of therapeutic practice (Porges, 2022; Dana, 2023).
- Everyday aesthetics: refers to the sensory and relational qualities of ordinary life, this includes but is not limited to the physicality, feel, and atmosphere of environments and interactions. In dementia care, everyday aesthetics might include places, spaces, relationships and objects an individual has access to, all of which can shape wellbeing (Saito, 2010; Leddy, 2015; Fox et al., 2025).
- Glimmers (polyvagal theory:) small cues that signal safety and connection to the nervous system, inviting calm, regulation, and belonging. A glimmer may be found in eye contact, a familiar song, environment, object, or the presence of a trusted person (Dana, 2024).
- Malignant social psychology: a term by Kitwood (1997) describing interactions that undermine personhood in dementia care, such as infantilisation, ignoring, or disempowerment. These practices contrast with positive person work, which affirms dignity and connection.
- Moments-based research: a strand of dementia studies that examines fleeting, micro-temporal interactions, such as humor, recognition, or shared silence which may be short-lived but carry deep personal significance and support a sense of self (Kindell et al., 2018; Keady et al., 2022; Dowlen et al., 2024).
- Positive person work: practices that affirm identity, autonomy, and dignity for people with dementia. This includes offering respect, recognition, and emotionally attuned interactions that sustain personhood (Kitwood, 1997)
- Triggers (polyvagal theory): sensory or relational cues that activate defensive autonomic states (fight, flight, freeze), often leading to distress, agitation, or withdrawal in people living with dementia (Porges, 2022; Dana, 2023).
